# Caspase-1-Dependent Pyroptosis of Peripheral Blood Mononuclear Cells Is Associated with the Severity and Mortality of Septic Patients

**DOI:** 10.1155/2020/9152140

**Published:** 2020-02-28

**Authors:** Yuchang Wang, Yukun Liu, Qinxin Liu, Qiang Zheng, Xijie Dong, Xinghua Liu, Wei Gao, Xiangjun Bai, Zhanfei Li

**Affiliations:** ^1^Trauma Center/Department of Emergency and Trauma Surgery, Tongji Hospital, Tongji Medical College of Huazhong University of Science and Technology, Wuhan 430030, China; ^2^Plastic and Aesthetic Surgery, Tongji Hospital, Tongji Medical College, Huazhong University of Science and Technology, Wuhan 430030, China

## Abstract

**Purpose:**

Pyroptosis has been known to play a vital role in the inflammation process which was induced by infection, injury, or inflammatory disease. The present study was aimed at evaluating the percentage of peripheral blood mononuclear cell (PBMC) pyroptosis in septic patients and assessing the correlation of PBMC pyroptosis with the severity and the mortality of septic patients.

**Methods:**

128 trauma-induced patients with sepsis were enrolled in this prospective cohort study. Blood samples were collected, and PBMC pyroptosis was measured by flow cytometry within 24 hours after sepsis was diagnosed.

**Results:**

Percentage of PBMC pyroptosis was positively correlated with the acute physiology and chronic health evaluation (APACHE) II score and sequential organ failure assessment (SOFA) score (all *P* < 0.01). The area under the curve (AUC) for the percentage of PBMC pyroptosis on a receiver operating characteristic curve was 0.79 (95% confidence interval (CI), 0.68–0.90). A Cox proportional hazard model identified an association between an increased percentage of PBMC pyroptosis (>14.17%) and increased risk of the 28-day mortality (hazard ratio = 1.234, 95% CI, 1.014–1.502).

**Conclusion:**

The percentage of PBMC pyroptosis increases in septic patients, and the increased percentage of PBMC pyroptosis is associated with the severity of sepsis and the 28-day mortality of patients with sepsis.

## 1. Introduction

The progression of trauma-induced sepsis leads to organ dysfunction and is a leading cause of death in severe trauma patients [1]. Currently, although the morbidity and mortality of sepsis have significantly decreased over the past few years, it remains difficult to treat [2, 3]. Rapid diagnosis and prompt intervention continue to be the primary treatments to reduce the mortality of sepsis.

It has been widely accepted that dysfunctional inflammatory reaction and bacterial clearance are the main mechanisms for the susceptibility to sepsis [4]. However, anti-inflammatory cytokine treatments are not expected as they were applied in clinical trials [5]. In recent years, researchers have paid more attention to the mechanism of immune cell death, which contributes to the dysregulated inflammatory reaction, immunosuppression, and organ failure in sepsis [6]. Pyroptosis is dependent on the activation of inflammatory caspases (i.e., caspase-1 and caspase-11 in mice and their orthologs caspase-1, caspase-4, and caspase-5 in humans), which can be triggered by various pathological stimuli [7, 8]. Unlike apoptosis, pyroptosis is a lytic and inflammatory mode of cell death and releases proinflammatory cytokines and danger signals into the extracellular matrix [7]. It is related to differential pathophysiological outcomes in infectious and chronic inflammatory diseases [9]. Furthermore, uncontrolled pyroptosis could become detrimental in the environment of autoinflammatory disease and sepsis [9].

Recently, many studies have focused on the complex roles of pyroptosis in inflammatory disease, including sepsis. According to several studies, the activation of pyroptosis has been found involved in multiple pathological conditions, including the identification of infection [10], the hereditary autoinflammatory syndromes [11], and the inflammatory bowel disease [12]. Furthermore, blocking pyroptosis signaling markedly reduces the organ damage and mortality in mice [13, 14]. However, there are rare clinical researches involving the role of pyroptosis in sepsis. In our previous studies, we found that pyroptosis of PBMCs was significantly increased and correlated with the severity of trauma. Meanwhile, pyroptotic PBMCs were a good marker to predict the development of sepsis in patients with severe trauma [15]. However, the correlation of pyroptotic PBMCs and prognosis of trauma-induced sepsis remains elusive.

## 2. Methods

### 2.1. Research Setting and Study Participants

This was a prospective cohort study of which patients and samples were collected from the Trauma Intensive Care Unit (TICU) of Tongji Hospital of the Tongji Medical College of Huazhong University of Science and Technology. The protocol was approved by the medical ethics committee of Tongji Hospital of the Tongji Medical College of Huazhong University of Science and Technology. All procedures were performed in accordance with the relevant guidelines and regulations. 145 consecutive patients over 18 years old were admitted to the TICU from March 2016 to August 2017. Among those patients, we selected a total of 128 trauma-induced septic patients which were defined by sepsis-3 [3]. Informed consents for the patients contributing to the samples were obtained. All septic patients were treated according to the guidelines of the Surviving Sepsis Campaign [16]. The exclusion criteria for patients included autoimmune disease, inherited or acquired immunodeficiency, long-term use of an immunosuppressive agent, acute myocardial infarction, or thromboembolic event.

### 2.2. Clinical Data Collection

The data of clinical characterization including demographic characteristics, vital signs, past medical history, laboratory examinations, image findings, diagnosis, and outcome were collected. The SOFA and APACHE II scores were also calculated during the first 24 hours after the patients were diagnosed with sepsis.

### 2.3. Blood Sampling and Isolation of PBMCs

Venous blood samples were collected in an EDTA vacutainer within 24 hours after patients were diagnosed with sepsis. PBMCs were isolated from the blood samples using density gradient centrifugation with Ficoll-Hypaque (TBD Science; Tianjin, China) according to the manufacturer's instruction.

### 2.4. Flow Cytometry

Pyroptosis of PBMCs was measured by flow cytometry (BD FACSCanto™ II; BD Biosciences, San Jose, CA, USA). Fluorescent-labelled inhibitors of caspase (FLICA) probe assays (ImmunoChemistry Technologies, Minneapolis, MN, USA) were performed to determine the pyroptosis according to the manufacturer's instruction. Pyroptotic PBMCs are distinctly immune-stained positive for FAM-FLICA-caspase-1 and PI. All flow cytometry assays were performed within 1 hour after blood was collected to ensure that the results are similar to that of in vivo condition.

### 2.5. Statistical Analysis

Data were described as percentages or median (95% confidence interval (CI)). Two groups were compared using the chi-squared test or Fisher's exact tests for categorical data and Student's *t-*test or analysis of variance (ANOVA) for continuous variables. Pearson correlation analyses were performed to estimate associations between pyroptotic PBMCs and the APACHE II or SOFA score. Receiver operating characteristic curves (ROCs) were established, and the area under the ROC curve (AUCs) was determined to evaluate the predictive qualities of pyroptotic PBMCs. Subsequently, the 28-day survivor and nonsurvivor groups were analyzed using a Cox proportional hazard model with a percentage of PBMC pyroptosis cut-off value to predict 28-day mortality according to the ROC and several variables. The Kaplan-Meier method was used to report survival curves that were analyzed using the log-rank test. The statistical analyses were conducted using GraphPad Prism 5.01 (GraphPad Software, Inc., La Jolla, CA, USA) or IBM SPSS version 23 (IBM Crop., Armonk, NY, USA). A *P* value of <0.05 was considered statistically significant.

## 3. Results

### 3.1. Demographic Characteristics of the Overall Study Population

**Figure 1:**
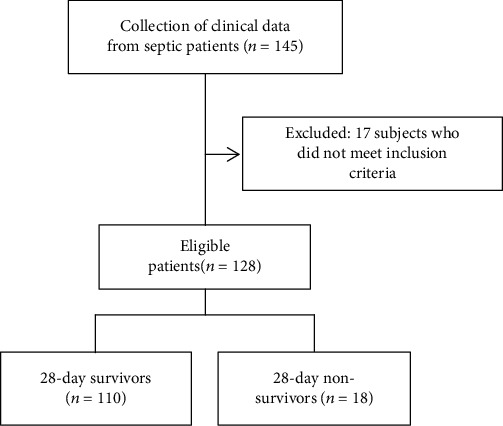
Flow diagram of the study population.

According to the inclusion criteria, 145 patients were admitted to our study. However, seven patients were excluded because of immunological disease and ten patients were excluded because of no blood sample within 24 hours after being diagnosed with sepsis. Therefore, a total of 128 septic patients were enrolled in the present study. There were 18 (14.06%) patients who died within the 28-day follow-up. The grouping method is shown in Figure 1. The average time of onset of sepsis after trauma is 6.82 days. The most common site of infection was the chest (65.63%), followed by the abdomen (18.75%). The cause of sepsis was bacterial infection in all cases. Ninety-seven Gram-positive and 39 Gram-negative bacteria were isolated, including 27 mixed bacterial infections. Fifty-eight positive blood cultures and 26 other site cultures (sputum, pleural fluid, ascites, urine, and pus) were identified (Table 1).

### 3.2. Comparison of Characteristics between the 28-Day Survivors and Nonsurvivors

**Figure 2:**
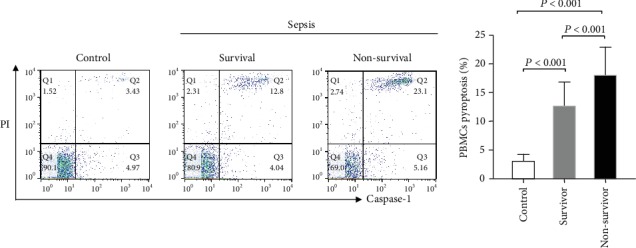
PBMC pyroptosis in healthy control subjects and septic patients (28-day survivors and nonsurvivors). Pyroptotic PBMCs are distinctly immune-stained positive for FAM-FLICA-caspase-1 and PI, as shown in area Q2.

Patients were divided into two groups according to the 28-day mortality. Ten age-matched and sex-matched healthy volunteers who have been confirmed with no clinical evidence of infection by physical examination were recruited as controls. There were no significant differences in age, gender, or correlative diseases among the patients in survivor or nonsurvivor groups. However, nonsurvivor patients had significantly higher APACHE II and SOFA scores than the survivor group (Table 2). In accordance with prior reports from our group, IL-6 and PCT were also markedly elevated in nonsurvivor patients (median IL-6 623.0 pg/ml and median PCT 2.36 pg/ml, respectively) as compared with survivor patients (median IL-6 332.8 pg/ml (*P* = 0.002) and median PCT 1.75 pg/ml (*P* = 0.02), respectively) (Table 2). Moreover, nonsurvivor patients had a higher proportion of pyroptotic PBMCs in their peripheral blood than the survivor group (survivor vs. nonsurvivor: 12.20% vs. 19.07%, *P* < 0.001) (Figure 2).

### 3.3. Correlations between PBMC Pyroptosis and Sepsis Severity

**Figure 3:**
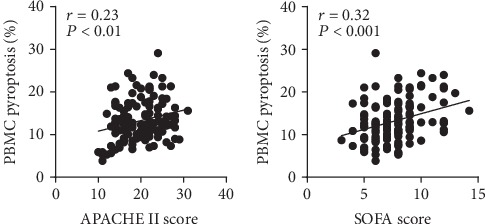
Positive correlations were observed between the percentages of pyroptotic PBMCs and the APACHE II and SOFA scores.

The Spearman correlation coefficient was used to assess the correlation between the percentage of pyroptotic PBMCs and the disease severity scoring systems (APACHE II and SOFA scores). Positive correlations were observed between the pyroptotic PBMC level and the APACHE II (*r* = 0.23, *P* < 0.01) and SOFA scores (*r* = 0.32, *P* < 0.01) (Figure 3).

### 3.4. Association between PBMC Pyroptosis and Mortality

**Figure 4:**
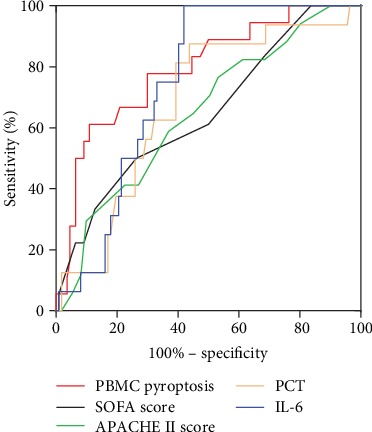
The ROC curves of PBMC pyroptosis, APACHE II score, SOFA score, PCT, and IL-6 for predicting 28-day mortality.

ROC curves for the PBMC pyroptosis, SOFA score, APACH II score, PCT, and IL-6 were constructed based on statistically significant differences, and areas under the curves (AUCs) were calculated. The AUCs for the APACHE II score, SOFA score, PCT, and IL-6 were 0.65 (95% CI, 0.51-0.78), 0.65 (95% CI, 0.51-0.79), 0.68 (95% CI, 0.55-0.81), and 0.75 (95% CI, 0.66-0.83), respectively. PBMC pyroptosis was better than any other indicator, with an AUC of 0.79 (95% CI, 0.68-0.90) (Table 3 and Figure 4).

**Figure 5:**
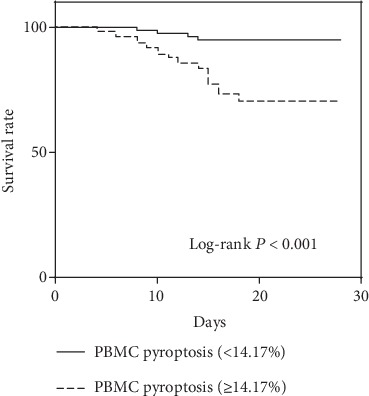
Kaplan-Meier survival analysis showed that the 28-day mortality of patients with percentage of PBMC pyroptosis ≥14.17% was higher than that of patients with percentage of PBMC pyroptosis <14.17%.

A cut-off value higher than 14.17% for pyroptotic PBMCs was determined to predict 28-day mortality on the ROC (sensitivity, 77.78%; specificity, 70.00%). Notably, the Kaplan-Meier survival curve showed that the patients with percentage of pyroptotic PBMCs above 14.17% were at greater risks of death than others (*P* < 0.001 by the log-rank test) (Figure 5). This indicated that higher percentages of pyroptotic PBMCs were associated with higher mortality of septic patients.

For further risk assessment, patients were divided into two groups according to the cut-off level of 14.17% and subjected to a Cox proportional hazard model analysis. In the univariate analysis, pyroptotic PBMCs, APACHE II score, and PCT were associated with the 28-day mortality. The hazard ratios (95% CI) for PBMC pyroptosis, APACHE II score, and PCT were 1.345 (1.157-1.564), 1.366 (1.124-1.660), and 2.673 (1.248-5.723), respectively. In the multivariate analysis, sex and age were not found to be associated with 28-day mortality. However, pyroptotic PBMCs remained significant with a hazard ratio of 1.234 (95% CI, 1.014-1.502) (Table 4).

## 4. Discussion

Sepsis, a fatal inflammatory syndrome associated with disseminated infection, can result in multiple organ failure [3]. It is widely accepted that the result of excessive and uncontrolled inflammation initiated an immune-suppressed status [5]. This latter status was thought to occur through a variety of detrimental factors that consequently lead to immune cell shortage or dysfunction. In recent years, more attention has been attracted to the role of pyroptosis of immune cells in immune disorder of sepsis [6, 17, 18]. In the current study, we found that the percentages of pyroptotic PBMCs in nonsurvivors were significantly higher than those of the survivors in trauma-induced sepsis. Furthermore, pyroptotic PBMCs, which correlate with sepsis severity and increased mortality, may serve as a good indicator to predict mortality in septic patients.

The APACHE II score and SOFA score are indicators which have been extensively used to evaluate the severity of sepsis patients [19, 20]. The higher APACHE II score and SOFA score usually relate to the increased mortality in sepsis [19, 21]. Our results indicated that PBMC pyroptosis positively correlated with the APACHE II score and SOFA score. Furthermore, the PBMC pyroptosis level was comparable to those scores with the ROC curve and Cox hazard proportional model. These results revealed that a certain proportion of PBMC pyroptosis level, which was above 14.17%, related to a significant increase in mortality (hazard ratio, 1.234).

Pyroptosis is an inflammatory programmed cell death and is dependent on the activation of inflammatory caspases [7, 8, 22]. Several previous studies have demonstrated that pyroptosis helps protect against invasive pathogens [23, 24]. However, pyroptotic cells release large amounts of inflammatory mediators (IL-18, IL-1*β*, HMGB1, etc.) as well as danger-associated molecular patterns (DAMPs) (such as ATP and uric acid), which could contribute to proinflammatory cascade reaction [7, 9, 25]. It is notable that excessive inflammation is risky which may result in organ injury and death in sepsis.

Accumulating studies have confirmed a relationship between pyroptosis and inflammation. IL-1*β* and IL-18 are typical products of pyroptosis and are usually secreted from plasma membrane pores of pyroptotic cells. IL-1*β*, a potent endogenous pyrogen, could stimulate fever, leukocyte tissue migration, and expression of diverse cytokines and chemokines [25]. IL-18 plays an important role in the activation of T cells, macrophages, and other immune cells [25, 26]. Also, it has been known that the neutralization of IL-1*β* and IL-18 simultaneously could completely protect against a lethal LPS challenge in septic mice [27]. In addition, the pyroptotic death of immune cells induced by diverse stimuli may contribute to cytopenia and immune suppression [28].

As mentioned previously, caspase-1-dependent pyroptosis plays an important role in the occurrence and development of sepsis. Numerous studies have demonstrated that specific inhibition of caspase-1 could decrease the bacterial load and inflammation and is beneficial to survival in mouse models of sepsis. In mouse models of sepsis, Wu et al. have certified that the use of a caspase-1 inhibitor, AC-YVAD-CMK, could alleviate the pyroptosis of alveolar macrophages and ALI [14]. Several studies also revealed that pyroptosis of the vascular endothelium played a critical role in the development of acute lung injury [29, 30]. Moreover, an animal model of lipopolysaccharide- (LPS-) induced acute liver injury has revealed that a caspase-1 inhibitor, AC-YVAD-CMK, could reduce pyroptosis-related inflammatory cytokines, IL-1*β* and IL-18, therefore relieving acute liver injury [13]. In the field of HIV, 95% quiescent lymphoid CD4^+^T cells died of caspase-1-mediated pyroptosis triggered by abortive viral infection and blocking CD4^+^T cell pyroptosis was considered a potential “anti-AIDS” therapy [31]. These findings suggest that the activation of pyroptosis has various adverse effects on the host immune system. In addition, we found that pyroptotic PBMCs significantly related to the prognosis of sepsis and could be a potential biomarker to predict the mortality of sepsis. In accordance with other studies, pyroptosis blockade could protect mice against infection and sepsis by alleviating inflammation, which may serve as a promising therapy target to alleviate MODS in sepsis patients.

Nevertheless, our study has some limitations. Initially, it is a single-center study and the sample size is relatively small. Furthermore, the percentage of pyroptotic PBMCs was detected at a single time point, and the dynamic change of pyroptotic PBMCs warrants further exploration. Additionally, PBMCs include subsets such as lymphocytes (T cells, B cells, and NK cells) and monocytes, which need to be investigated whether they are involved in pyroptosis in sepsis. Finally, this study is only an observational research and further studies are required to explore the molecular mechanisms of our findings.

## 5. Conclusion

This study provides new insight that the percentage of PBMC pyroptosis increases in septic patients. Moreover, an increased percentage of PBMC pyroptosis is associated with the severity of sepsis and the 28-day mortality among septic patients.

## Figures and Tables

**Table 1:** Characteristics of septic patients.

Characteristic	Sepsis (*n* = 128)
Age (years)	47.53 (36.33-52.79)
Male, *n* (%)	92 (71.88)
Time period of onset of sepsis after trauma (d)	6.82 (4.17-8.26)
Injury mechanism, *n* (%)	
Motor vehicle collision	98 (76.56)
Fall	18 (14.06)
Others	12 (9.38)
Site of infection, *n* (%)	
Chest	84 (65.63)
Abdomen	24 (18.75)
Soft tissue	9 (7.03)
Urinary	6 (4.69)
Others	5 (3.91)
Microbial data, *n* (%)	
Gram positive	97 (75.78)
Gram negative	39 (30.47)
Mixed	27 (21.09)
Positive blood cultures	58 (45.31)
Other site cultures	26 (20.31)

Values are expressed as *n* (%) or median (95% confidence interval) unless otherwise indicated.

**Table 2:** Comparison of characteristics between 28-day survivors and 28-day nonsurvivors.

Variable	Survivors (*n* = 110)	Nonsurvivors (*n* = 18)	*P*
Gender (male), % (*n*)	71.82 (79)	72.22 (13)	0.99
Age (year)	47.60 (45.18-50.02)	48.75 (41.83-55.67)	0.55
APACHE II score	20.00 (18.54-20.34)	22.00 (20.43-24.40)	0.01
SOFA score	7.50 (7.15-7.93)	8.50 (7.64-9.92)	0.04
Severe sepsis or septic shock, % (*n*)	20.00 (22)	61.11 (11)	<0.001
Underlying disease, % (*n*)			
Hypertension	20.91 (23)	22.22 (4)	0.99
Diabetes	8.18 (9)	16.67 (3)	0.37
Infection site, % (*n*)			
Respiratory	66.36 (73)	61.11 (11)	0.79
Abdomen	19.09 (21)	16.67 (3)	0.99
Urinary tract	4.55 (5)	5.56 (1)	0.99
Blood	43.64 (48)	55.56 (10)	0.45
Others	3.64 (4)	5.56 (1)	0.54
Laboratory parameters			
WBC (×10^9^)	13.64 (9.78-15.28)	15.49 (10.46-18.77)	0.11
LC (×10^9^)	0.79 (0.74-0.84)	0.61 (0.49-0.72)	0.003
CRP (pg/ml)	67.05 (60.39-73.71)	84.80 (62.52-107.1)	0.13
PCT (pg/ml)	1.75 (1.53-1.96)	2.36 (1.85-2.86)	0.02
IL-6 (pg/ml)	332.8 (262.9-402.6)	623.0 (460.9-785.0)	0.002
IL-1*β* (pg/ml)	18.83 (16.67-20.99)	23.63 (15.45-31.80)	0.25
IL-18 (pg/ml)	420.4 (354.3-486.6)	693.8 (568.4-819.3)	<0.001

Values are presented as median (95% confidence interval (CI)) or percentage (%). APACHE: acute physiology and chronic health evaluation; SOFA: sequential organ failure assessment; LC: lymphocyte count; WBC: white blood cells; CRP: C-reactive protein; PCT: procalcitonin.

**Table 3:** Areas under the ROC curves for predicting 28-day mortality in septic patients.

Parameter	AUC	Sensitivity (%)	Specificity (%)	95% CI	*P*
PBMC pyroptosis	0.79	77.78	70.00	0.68-0.90	<0.01
SOFA score	0.65	50.00	73.64	0.51-0.79	0.04
APACHE II score	0.65	76.47	46.85	0.51-0.78	0.05
PCT	0.68	87.50	56.25	0.55-0.81	0.02
IL-6	0.75	75.00	66.96	0.66-0.83	<0.01

PBMCs: peripheral blood mononuclear cells; SOFA: sequential organ failure assessment; APACHE: acute physiology and chronic health evaluation; PCT: procalcitonin; IL: interleukin; AUC: area under the curve; CI: confidence interval.

**Table 4:** Cox proportional hazard model of PBMC pyroptosis and 28-day mortality.

Variable	Univariate analysis			Multivariate analysis		
	HR	95% CI	*P*	HR	95% CI	*P*
Sex (age)	0.686	0.240-1.957	0.481			
Age (year)	0.978	0.944-1.014	0.224			
APACHE II score	1.366	1.124-1.660	0.002	1.148	0.903-1.458	0.259
Lymphocyte count	3.731	0.178-78.132	0.396			
PBMC pyroptosis	1.345	1.157-1.564	≤0.001	1.234	1.014-1.502	0.036
IL-18	1.002	1.000-1.004	0.088			
IL-6	1.000	0.999-1.002	0.675			
PCT	2.673	1.248-5.723	0.011	1.828	0.753-4.438	0.182

APACHE: acute physiology and chronic health evaluation; PBMCs: peripheral blood mononuclear cells; IL: interleukin; PCT: procalcitonin.

## Data Availability

The data used to support the findings of this study are available from the corresponding author upon request.

## Abbreviations

PBMCs: Peripheral blood mononuclear cells
IL: Interleukin
SOFA: Sequential organ failure assessment
APACHE: Acute physiology and chronic health evaluation
PCD: Programmed cell death
CI: Confidence interval
ANOVA: Analysis of variance
FLICA: Fluorescent-labelled inhibitors of caspase
ROC: Receiver operating characteristic
AUC: Area under the curve
LC: Lymphocyte count
TNF: Tumour necrosis factor
GSDMD: Gasdermin D
TICU: Trauma Intensive Care Unit
CRP: C-reactive protein
PCT: Procalcitonin.

## Additional Points

*Study Site*. All patients were enrolled at Tongji Hospital, Tongji Medical College of Huazhong University of Science and Technology, 1095 Jiefang Avenue, Wuhan, Hubei Province, China.

## References

[1] Dewar D., Moore F. A., Moore E. E., Balogh Z. (2009). Postinjury multiple organ failure. *Injury*.

[2] Angus D. C., van der Poll T. (2013). Severe sepsis and septic shock. *The New England Journal of Medicine*.

[3] Singer M., Deutschman C. S., Seymour C. W. (2016). The third international consensus definitions for sepsis and septic shock (sepsis-3). *Journal of the American Medical Association*.

[4] Rimmelé T., Payen D., Cantaluppi V. (2016). Immune cell phenotype and function in sepsis. *Shock*.

[5] Hattori Y., Hattori K., Suzuki T., Matsuda N. (2017). Recent advances in the pathophysiology and molecular basis of sepsis-associated organ dysfunction: novel therapeutic implications and challenges. *Pharmacology & Therapeutics*.

[6] Aziz M., Jacob A., Wang P. (2014). Revisiting caspases in sepsis. *Cell Death & Disease*.

[7] Vande Walle L., Lamkanfi M. (2016). Pyroptosis. *Current Biology*.

[8] Shi J., Gao W., Shao F. (2017). Pyroptosis: gasdermin-mediated programmed necrotic cell death. *Trends in Biochemical Sciences*.

[9] Man S. M., Karki R., Kanneganti T. D. (2017). Molecular mechanisms and functions of pyroptosis, inflammatory caspases and inflammasomes in infectious diseases. *Immunological Reviews*.

[10] Fink S. L., Cookson B. T. (2007). Pyroptosis and host cell death responses during Salmonella infection. *Cellular Microbiology*.

[11] Simon A., van der Meer J. W. (2007). Pathogenesis of familial periodic fever syndromes or hereditary autoinflammatory syndromes. *American Journal of Physiology Regulatory, Integrative and Comparative Physiology*.

[12] Siegmund B., Lehr H. A., Fantuzzi G., Dinarello C. A. (2001). IL-1 beta-converting enzyme (caspase-1) in intestinal inflammation. *Proceedings of the National Academy of Sciences of the United States of America*.

[13] Chen Y. L., Xu G., Liang X. (2016). Inhibition of hepatic cells pyroptosis attenuates CLP-induced acute liver injury. *American Journal of Translational Research*.

[14] Wu D. D., Pan P. H., Liu B. (2015). Inhibition of alveolar macrophage pyroptosis reduces lipopolysaccharide-induced acute lung injury in mice. *Chinese Medical Journal*.

[15] Wang Y., Liu Q. X., Liu T. (2018). Caspase-1-dependent pyroptosis of peripheral blood mononuclear cells predicts the development of sepsis in severe trauma patients: a prospective observational study. *Medicine*.

[16] Petros S., John S. (2017). The 2016 Surviving Sepsis Campaign sepsis guideline. *Medizinische Klinik - Intensivmedizin und Notfallmedizin*.

[17] Chen L., Zhao Y., Lai D. (2018). Neutrophil extracellular traps promote macrophage pyroptosis in sepsis. *Cell Death & Disease*.

[18] Hu Z. S., Murakami T., Suzuki K. (2016). Antimicrobial cathelicidin peptide LL-37 inhibits the pyroptosis of macrophages and improves the survival of polybacterial septic mice. *International Immunology*.

[19] Minne L., Abu-Hanna A., de Jonge E. (2008). Evaluation of SOFA-based models for predicting mortality in the ICU: a systematic review. *Critical Care*.

[20] Knaus W. A., Draper E. A., Wagner D. P., Zimmerman J. E. (1985). APACHE II: a severity of disease classification system. *Critical Care Medicine*.

[21] Sadaka F., Cytron M., Fowler K., Javaux V., O’Brien J. (2014). 996. *Critical Care Medicine*.

[22] Liang H., Liu Y. (2016). Gasdermins pore cell membrane to pyroptosis. *Science China Life Sciences*.

[23] Jorgensen I., Miao E. A. (2015). Pyroptotic cell death defends against intracellular pathogens. *Immunological Reviews*.

[24] Miao E. A., Leaf I. A., Treuting P. M. (2010). Caspase-1-induced pyroptosis is an innate immune effector mechanism against intracellular bacteria. *Nature Immunology*.

[25] Jorgensen I., Lopez J. P., Laufer S. A., Miao E. A. (2016). IL-1*β*, IL-18, and eicosanoids promote neutrophil recruitment to pore-induced intracellular traps following pyroptosis. *European Journal of Immunology*.

[26] Nakanishi K., Yoshimoto T., Tsutsui H., Okamura H. (2001). Interleukin-18 regulates both TH1 and TH2 responses. *Annual Review of Immunology*.

[27] Berghe T. V., Demon D., Bogaert P. (2014). Simultaneous targeting of IL-1 and IL-18 is required for protection against inflammatory and septic shock. *American Journal of Respiratory and Critical Care Medicine*.

[28] Croker B. A., O'Donnell J. A., Gerlic M. (2014). Pyroptotic death storms and cytopenia. *Current Opinion in Immunology*.

[29] Lai D. M., Tang J., Chen L. S. (2018). Group 2 innate lymphoid cells protect lung endothelial cells from pyroptosis in sepsis. *Cell Death & Disease*.

[30] Cheng K. T., Xiong S., Ye Z. (2017). Caspase-11-mediated endothelial pyroptosis underlies endotoxemia-induced lung injury. *The Journal of Clinical Investigation*.

[31] Doitsh G., Galloway N. L., Geng X. (2014). Cell death by pyroptosis drives CD4 T-cell depletion in HIV-1 infection. *Nature*.

